# OsYSL16 plays a role in the allocation of iron

**DOI:** 10.1007/s11103-012-9930-1

**Published:** 2012-05-29

**Authors:** Yusuke Kakei, Yasuhiro Ishimaru, Takanori Kobayashi, Takashi Yamakawa, Hiromi Nakanishi, Naoko K. Nishizawa

**Affiliations:** 1 Graduate School of Agricultural and Life Sciences, The University of Tokyo, 1-1-1 Yayoi, Bunkyo-ku, Tokyo, 113-8657 Japan; 2 Research Institute for Bioresources and Biotechnology, Ishikawa Prefectural University, 1-308 Suematsu, Nonoichi-machi, Ishikawa, 921-8836 Japan

**Keywords:** Iron transporter, Phytosiderophore, Deoxymugineic acid, YSL, Unelongated node

## Abstract

Graminaceous plants acquire iron by secreting mugineic acid family phytosiderophores into the rhizosphere and taking up complexes of iron and phytosiderophores through YSL (yellow stripe 1-like) transporters. Rice OsYSL15 is a transporter of the iron(III)-2′-deoxymugineic acid complex. OsYSL16 has 85 % similarity to both OsYSL15 and the iron(II)-nicotianamine transporter OsYSL2. In the present study, we show that OsYSL16 functionally complemented a yeast mutant defective in iron uptake when grown on medium containing iron(III)-deoxymugineic acid, but not when grown on medium containing iron(II)-nicotianamine. *OsYSL16*-knockdown seedlings were smaller than wild-type seedlings when only iron(III)chloride was supplied as an iron source. The iron concentration in shoots of *OsYSL16*-knockdown plants was similar to that of the wild type; however, they showed more severe chlorosis than wild-type plants under iron-deficient conditions. Furthermore, *OsYSL16*-knockdown plants accumulated more iron in the vascular bundles of the leaves. Expression of the *OsYSL16* promoter fused to the *β*-glucuronidase gene showed that *OsYSL16* is expressed in the root epidermis and vascular bundles of whole plants. The expression was typically observed around the xylem. In the vascular bundles of unelongated nodes, it was detected in the xylem of old leaves and the phloem of new leaves. Graminaceous plants translocate iron from the roots to old leaves mainly via the xylem and to new leaves mainly via the phloem. Our results suggest that OsYSL16 plays a role in the allocation of iron(III)-deoxymugineic acid via the vascular bundles.

**Electronic supplementary material:**

The online version of this article (doi:10.1007/s11103-012-9930-1) contains supplementary material, which is available to authorized users.

## Introduction

Iron (Fe), an essential nutrient for all living organisms, is required for electron transfer during respiratory and photosynthetic reactions in mitochondria and chloroplasts, respectively (Marschner 1995). Fe deficiency is a major agricultural problem and contributes to reduced crop yields (Wallace and Lunt 1960). Fe deficiency anemia is among the most widespread dietary concerns in humans (WHO 2002). As plants are a primary source of food, it is important to understand the mechanisms of Fe uptake and translocation in plants for both agricultural and human health purposes.

Although Fe is the fourth most common element in the Earth’s crust, its bioavailability for plants is low. Fe is present mainly as insoluble Fe(OH)_3_ in aerobic soils. The solubility of Fe(OH)_3_ depends on the pH of the soil; the solubility is 10^−8^ M at pH 4 and 10^−17^ M at pH 7. Plants demand between 10^−8^ M and 10^−5^ M Fe and therefore face Fe deficiency in alkaline soils. Two distinct strategies for Fe uptake help to overcome the shortage of bioavailable Fe (Römheld and Marschner 1986).

Under Fe-deficient conditions, graminaceous plants secrete mugineic acid family phytosiderophores (MAs) into the rhizosphere (Takagi 1976; Takagi et al. 1984), and MAs solubilize Fe by forming Fe(III)-MAs complexes. Recently, the efflux transporter of MAs, TOM1 (Nozoye et al. 2011) was identified from rice secreting 2′-deoxymugineic acid (DMA). Graminaceous plants take up Fe(III)-MAs through ZmYS1 (Curie et al. 2001) and YSL (YS1-like) family transporters. *ZmYS1* was identified in the Fe-deficient mutant *ys1* of *Zea mays* (von Wirén et al. 1994). The Fe uptake system using MAs is referred to as strategy II. Among the strategy II plants, rice uses OsIRT1 to take up Fe(II), which is abundant in submerged fields (Ishimaru et al. 2006). Other types of plants employ strategy I for Fe uptake. For example, *Arabidopsis thaliana* exports protons and secretes phenolics and other compounds to solubilize Fe (Römheld and Marschner 1986). Fe(III) is reduced to Fe(II) by the ferric-chelate reductases FRO2 (Robinson et al. 1997, 1999). Subsequently, Fe(II) is absorbed by the Fe-regulated transporters IRT1 (Eide et al. 1996) and IRT2 (Vert et al. 2001).

ZmYS1 transports metal-MAs and metal-nicotianamine (NA) complexes (Curie et al. 2001; Schaaf et al. 2004). Barley *HvYS1* is expressed in the roots and transports Fe(III)-MA (Murata et al. 2006). Rice has 18 YSL genes. Rice *OsYSL15* is expressed in the roots and takes up Fe(III)-DMA from the rhizosphere (Inoue et al. 2009; Lee et al. 2009). *OsYSL2* is expressed in the vascular bundles and transports Fe(II)-NA and Mn(II)-NA (Ishimaru et al. 2010), making it important for the translocation of Fe and Mn. OsYSL18, which is expressed in pollen and is important for reproduction, transports Fe(III)-DMA (Aoyama et al. 2009). Strategy I plants also possess YSL transporters. TcYSL3 transports Fe(II)-NA and Ni–NA in *Thlaspi caerulescens* (Gendre et al. 2007). In *Arabidopsis thaliana*, AtYSL2 is associated with the translocation of Fe and Zn (Schaaf et al. 2005), and AtYSL1 and AtYSL3 are related to the translocation of Fe, Zn, Mn, and Cu (Waters et al. 2006). Based on the YSL transporter phylogenetic tree, the OsYSL transporter family can be divided into four subgroups (Yordem et al. 2011). OsYSL16, OsYSL15, OsYSL2, and ZmYS1 belong to the same subgroup. Gene expression of *OsYSL15* and *OsYSL2* is induced strongly by Fe deficiency (Koike et al. 2004; Inoue et al. 2009; Lee et al. 2009), whereas expression of *OsYSL16* is either constitutive or weakly induced in Fe-deficient roots depending on growth conditions (Koike et al. 2004; Inoue et al. 2009; Lee et al. 2009). Very recent report by Lee et al. (2012) indicated that activation of *OsYSL16* enhances Fe efficiency by facilitating Fe distribution within a plant, but precise function and transport activity of OsYSL16 is still unknown. In the present report, we analyzed the function of OsYSL16 more precisely in Fe uptake and translocation. Yeast complementation assay, tissue localization of *OsYSL16* expression and Fe-inefficient phenotype of *OsYSL16* knockdown plants indicated that OsYSL16 is a functional Fe(III)-DMA transporter responsible for Fe allocation via the vascular bundles, especially xylem.

## Materials and methods

### Construction of vectors for yeast complementation assay

Two vectors, pDR195 and pDR196, were used for the yeast complementation assay. Only the multiple-cloning sites differ between these vectors, and both have been successfully used in previous complementation assays of OsYSL15 and ZmYS1 (Inoue et al. 2009; Rentsch et al. 1995; Schaaf et al. 2004, 2005). For the expression of OsYSL16, pDR195 was used, and pDR196 was used for the expression of ZmYS1. *OsYSL16* cDNA was subcloned from a cDNA library of Fe-deficient rice roots (Koike et al. 2004), excised using *Not*I and *Xho*I, and inserted into the multicloning site of pDR195, to form pDR195-OsYSL16. Empty pDR195 vector was used as a negative control.

### Yeast complementation

A mutant strain of *Saccharomyces cerevisiae* that was defective in Fe uptake was used in this study; it was designated as *Δfet3Δfet4*: DEY1453 (MATα/MATα ade2/+canl/canl his3/his3 leu2/leu2 trpl/trpl ura3/ura3 fet3-2::HIS3/fet3-2::HIS3fet4-1::LEU2/fet4-1::LEU2; Dix et al. 1994). Yeast cells were grown in YPD (1 % yeast extract, 2 % peptone, and 2 % glucose, pH 4.0) or SD medium containing URA and TRP (pH 5.0). Agar was added at 2 % for solid plate medium. The Fe(III)-DMA complexes were prepared as described previously (20 mM FeCl_3_ and 100 mM DMA; Schaaf et al. 2005). The Fe(II)-NA was prepared by mixing appropriate amounts of 10 mM FeCl_2_ (pH 2), MES/Tris buffer (pH 7.0), and 100 mM NA for 2 h at room temperature, followed by filtration through an Amicon Ultrafree MC 0.22-μm filter unit (Millipore) to remove invisible but possible fraction of oxidized and precipitated Fe. The three vectors, pDR195-OsYSL16, pDR196-ZmYS1, and empty pDR195, were introduced into the Δfet3Δfet4 strain using the lithium acetate transformation method (Gietz and Schiestl 1995). For complementation assays, single colonies of transformed yeast cells were cultured in liquid YPD medium (pH 4.0). The cells were washed twice in 1 ml of 10 mM Tris containing 500 mM EDTA (pH 7.0) before each incubation period. The Fe(III)-DMA medium contained 20 μM FeCl_3_ and 100 μM DMA; the Fe(II)-NA medium, 10 μM FeCl_2_ and 100 μM NA; the no Fe medium, 0 μM FeCl_3_; and the control Fe medium, 20 μM FeCl_3_. The OD_660_ of the cultures was adjusted to 1.0, and 8 μl of 10× serial dilutions of the cultures were spotted onto SD plates to test for complementation.

### Subcellular localization of OsYSL16-green fluorescent protein (GFP)

The open reading frame (ORF) of OsYSL16 was amplified using the primers OsYSL16GFP.Fw (5′-CACCATGGACCGCCACGCGCTGGGCGGCG-3′) and OsYSL16GFP.Rv (5′-GTTTCCCGGTATGAACTTCATGCAG-3′). The amplified fragment was subcloned into pENTR/D-TOPO (Invitrogen), and this entry vector was designated as pENTR-OsYSL16. Using pDEST35S-sGFP (Ishimaru et al. 2005) as the destination vector, LR recombination reactions (Invitrogen) between the destination and entry vectors generated an expression clone containing the gene encoding 35S-OsYSL16-sGFP. Onion (*Allium cepa* L.) epidermal cells were transformed using a Biolistic PDS-1000/He particle delivery system (Bio-Rad), and the transiently expressed sGFP fluorescence was observed using a laser-scanning confocal microscope (LSM510, Karl Zeiss) according to Mizuno et al. (2003). Cells were stained with 2 μM FM4-64 immediately before imaging.

### OsYSL16 promoter-GUS analysis

The 1.5-kb 5′-upstream region of the OsYSL16 gene (−1,500 to −1 bp from the putative translation initiation codon) was amplified by PCR using genomic DNA as a template and the following primer set: OsYSL16GUS.Fw (5′-GAGAGAAAGCTTATGGACAACTGATAAGGCTCTTTTTTCT-3′) and OsYSL16GUS.Rv (5′-GAGAGAGCTAGCGCTGCCGGCCGCACCCGACGAGGCGC-3′). The amplified and verified fragment was excised with *Hind*III and *Nhe*I, and subcloned upstream of the *uidA* ORF, which encodes beta-D-glucuronidase (GUS), in pIG121Hm vector (Hiei et al. 1994). An *Agrobacterium tumefaciens* strain (C58) carrying the above construct was used to transform rice (*O. sativa* L. cv. Tsukinohikari) as described by other investigators (Higuchi et al. 2001). GUS expression in T1 plants was analyzed as described previously (Inoue et al. 2003). Histochemical staining during seed germination was performed as described elsewhere (Nozoye et al. 2007).

### Generation and characterization of OsYSL16 knockdown rice

To suppress *OsYSL16* expression, a 300-bp fragment of the *OsYSL16* gene was amplified by PCR with the primers 5′-CACCCCGGGAAACTAGCCAATGCAT-3′ and 5′-GCCAATAAGCACAAGTAATTG-3′. The amplified fragment was subcloned into a Gateway pENTR/D-TOPO cloning vector (Invitrogen). Using an LR clonase reaction (Invitrogen), the verified fragment was transferred into pIG121-RNAi-DEST (Ogo et al. 2007) and used for rice (cv. Nipponbare) transformation.

### Quantitative RT-PCR analysis

Quantitative RT-PCR analysis of seedlings was performed as described previously (Inoue et al. 2008) using the primers 5′-TCGCGCTGGCCAAGGTCAAGCCACC-3′ and 5′-ATGCATTCTAGAGTTTCCCGGTATGAACT-3′. A plasmid containing a full-length cDNA clone (AK070304, provided by the National Institute of Agrobiological Sciences, Japan) was used to check the copy number of *OsYSL16*. The primers used to amplify the other YSL genes were as described previously (*OsYSL15*, Inoue et al. 2008; *OsYSL2*, Ishimaru et al. 2010; and *OsYSL18*, Aoyama et al. 2009). For quantitative RT-PCR analysis, seeds were germinated on MS medium for 7 days.

### Plant materials

Wild-type (cv. Nipponbare) and transgenic rice seeds were germinated on MS medium and transferred to nutrient solution (100 μM Fe(III)-EDTA, Higuchi et al. 1995) in a greenhouse with 30 °C-light/25 °C-dark periods under natural light conditions. Fifteen plants were cultured in 20-l plastic boxes covered with plastic boards. The pH of the culture solution was adjusted daily to 5.3 with 1 M HCl. To determine SPAD values and shoot and root lengths, 3-week-old plants were transferred to nutrient solution without Fe and grown for another week. For Fe allocation analysis, 5-week-old plants were transferred to nutrient solution without Fe and grown for another week. Plants for Fe allocation analysis were separated into root and aerial parts. The aerial part was separated again into unelongated nodes and the shoot at 1 cm from the root. The shoot was separated into leaf blades and sheaths of both new (first- and second-newest) and old (third-, fourth- and fifth-newest) leaves.

### Growth assay during the germination stage

Non-transgenic (cv. Nipponbare) and OsYSL16-knockdown rice seeds were germinated and grown for 6 days on MS medium and on MS medium without Fe, Zn, Cu, or Mn. These plants were also germinated on MS medium in which the Fe source (100 μM Fe(III)-EDTA) was replaced by 100 μM Fe(III)-citrate, 10 μM Fe(II)Cl_2_, or 10 μM Fe(III)Cl_3_.

### Measurement of metal concentrations

Concentrations of metals were measured as described previously (Masuda et al. 2009) using ICP-AES (SPS1200VR; Seiko). Plant materials were dried at 80 °C for 12 h and weighed. Shoots and roots were digested separately in 2 ml of 11 N HNO_3_ for 20 min at 240 °C using a MARS XPRESS microwave system (CEM, Matthews, NC, USA). Leaf sheaths and unelongated nodes were digested for 60 min at 240 °C.

### Prussian blue staining

Non-transgenic (cv. Nipponbare) and OsYSL16-knockdown rice seeds were germinated and grown for 7 days on MS medium and seedlings from each line were transferred to 3.5-CL pots containing artificial soil [a 2:1 mixture of bonsol-ichigou (Sumitomo chemical, Japan) and vermiculite]. The soil was fertilized evenly with 2 g of LongTotal-70 (Chisso Asahi, Japan) per plant. The plants were grown in a greenhouse under natural light, with 14 h of light at 30 °C and 10 h of dark at 25 °C. The most expanded leaves of 3-week-old plants were sampled and soaked in 100 % ethanol for 24 h. The samples were soaked in 2 % HCl (Wako) and 2 % potassium hexacyanoferrate (II) trihydrate (Wako) for 24 h. Stained samples were washed with distilled water.

## Results

### Yeast complementation assay

**Fig. 1:**
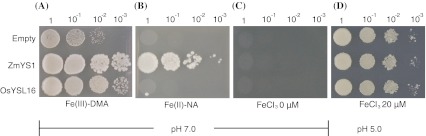
Yeast complementation assay. The vectors pDR195 (Empty), pDR196-ZmYS1 (ZmYS1), and pDR195-OsYSL16 (OsYSL16) were introduced respectively into the yeast strain Δ*fet3*Δ*fet4*. Each strain was grown on SD medium. **a** Fe(III)-DMA (pH 7.0): 20 μM FeCl_3_ with 100 μM DMA. **b** Fe(II)-NA (pH 7.0): 10 μM FeCl_2_ with 100 μM NA. **c** FeCl_3_, 0 μM (pH 7.0). **d** FeCl_3_, 20 μM (pH 5.0). The yeast were washed with EDTA, and water was added to adjust the OD_660_ to 1.0, 0.1, 0.01, and 0.001 (from the *left*) before spotting

Transport activity of OsYSL16 was investigated by complementation assay using the yeast Δ*fet3*Δ*fet4* mutant, which is defective in Fe uptake (Dix et al. 1994). ZmYS1 was used as a positive control, because it rescues the growth defect of the Δ*fet3*Δ*fet4* mutant when supplemented with either Fe(III)-DMA or Fe(II)-NA (Schaaf et al. 2004). Similarly to ZmYS1, OsYSL16 rescued the growth defect of the Δ*fet3*Δ*fet4* mutant on medium containing Fe(III)-DMA, but the control vector did not (Fig. 1a). Although ZmYS1 also rescued the growth defect on medium containing Fe(II)-NA, neither OsYSL16 nor the control vector did (Fig. 1b). No colonies survived on medium without Fe (Fig. 1c). The effects of OsYSL16, ZmYS1, and the control vector did not differ on growth medium with 20 μM FeCl_3_ (Fig. 1d).

The subcellular localization of OsYSL16 was assessed using OsYSL16-GFP fusion protein expressed in onion cells. The fluorescence of OsYSL16-GFP co-localized primarily with the plasma membrane marker FM4-64 (supplementary Fig. 1), in accordance with the plasma membrane localization reported by Lee et al. (2012).

### Localization of OsYSL16 promoter-GUS expression in roots, shoots, and seedlings

**Fig. 2:**
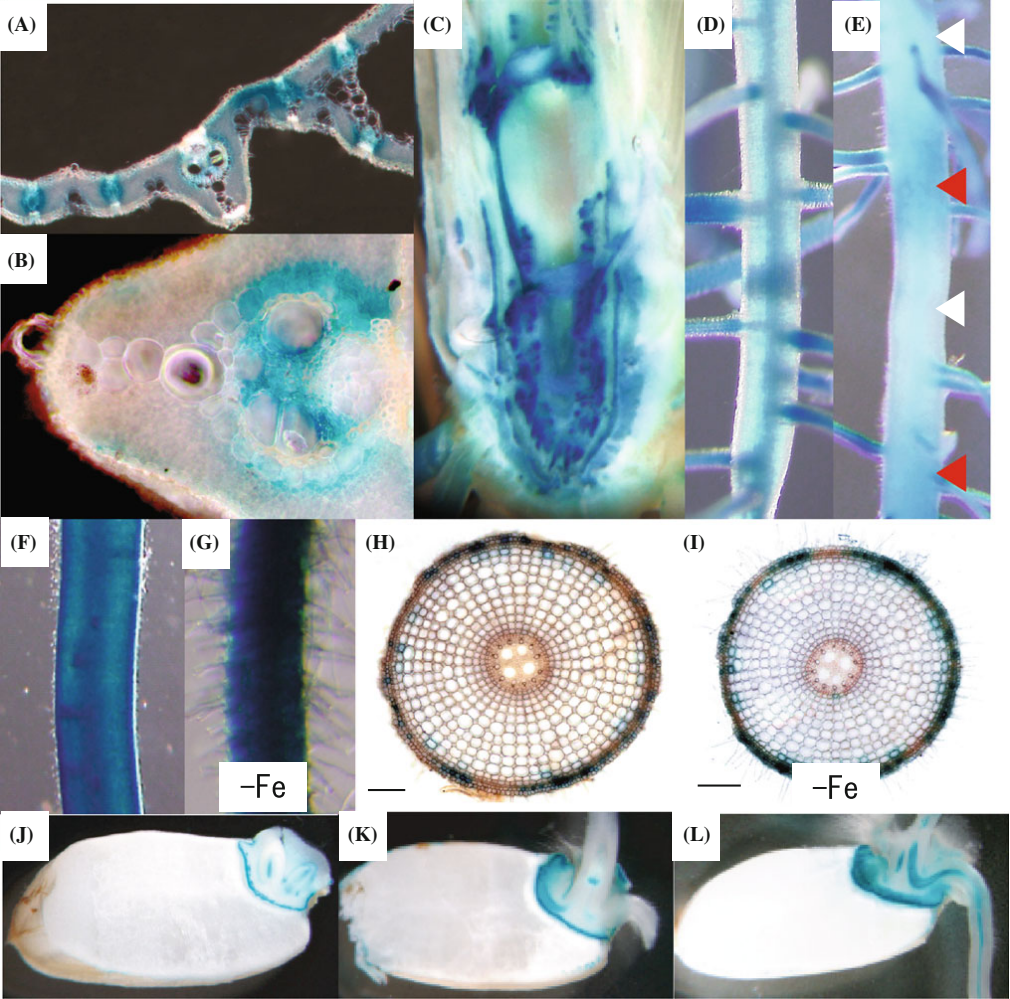
Tissue localization of OsYSL16. **a** *OsYSL16* promoter-GUS activity in leaf blades. **b** Large vascular bundle of the leaf. **c** Unelongated node. **d** Part of a root not showing a striped pattern. **e** Part of a root showing a striped pattern. **f**, **g** Main root. **h**, **i** Horizontal root section. Images g and i are from a plant grown under Fe-deficient conditions (–Fe), and others represent growth under normal conditions. Scale bars are 200 μm. **j** Seeds 1 day after germination. **k** Seeds 2 days after germination. **l** Seeds 3 days after germination

To investigate the tissue localization of OsYSL16, its promoter was fused with the *uidA* gene and introduced into rice. GUS activity was analyzed 3 weeks after germination. GUS activity was detected in leaves, unelongated nodes, and roots (Fig. 2). In leaves, GUS activity was observed mainly in the vascular bundles under Fe-sufficient conditions (Fig. 2a), and this was unchanged under Fe-deficient conditions (data not shown). GUS activity was relatively strong around xylem vessels (Fig. 2b). In unelongated nodes, strong GUS activity was observed in most vascular bundles (Fig. 2c). Further details regarding the activity in unelongated nodes are provided in the section below. In the root, the localization of GUS activity was not constant. Activity was observed mainly in the vascular bundles and at main root-lateral root junctions (Fig. 2d); it sometimes exhibited a striped pattern, with alternately strong and weak staining (red and white triangles in Fig. 2e). The activity under Fe-deficient conditions in root epidermis and root hair was largely similar to that under Fe-sufficient conditions, but more cells showed GUS activity with the increase in root hairs (Fig. 2f–i). In a horizontal root section, activity was detected only in some epidermal cells under both Fe-sufficient (Fig. 2h) and Fe-deficient conditions (Fig. 2i). Root hairs that formed under Fe-deficient conditions showed activity (Fig. 2g, i). During germination, GUS activity was observed in the vascular bundles and scutellum (Fig. 2j–l). No activity was detected in the endosperm.

### Localization of OsYSL16 promoter-GUS expression in unelongated nodes

**Fig. 3:**
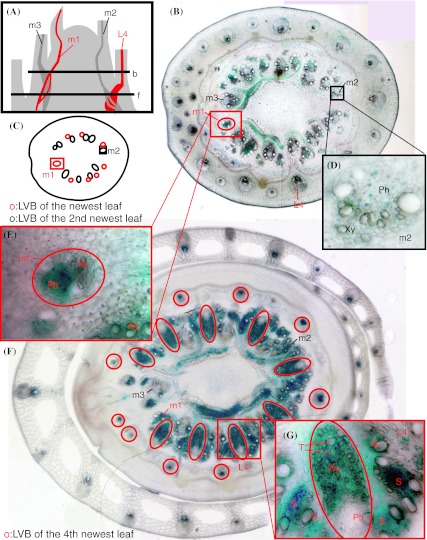
Localization of OsYSL16 in unelongated nodes. **a** Vertical schematic of unelongated nodes, midveins, LVBs, and sections **b** and **f**. **b** Picture of section **b**. **c** Model of LVBs of the newest and second-newest leaves in section **b**. **d** Magnified view around the midvein of the second-newest leaf. **e** Magnified view around the midvein of the newest leaf. **f** Picture of section **f**. **g** Magnified view of extended LVB in section **f**. LVB, Large vascular bundle; *Ph* phloem, *Xy* xylem, *T* tranched, *S* small vascular bundle, *m1–m4* midveins of the first-through fourth-newest leaves, *L4* LVB of the fourth-newest leaf

GUS activity was very strong in the vascular bundles of unelongated nodes (Fig. 2c), with expression in the upper parts of unelongated nodes where large vascular bundles (LVBs) of the newest leaf and the second-, third-, and fourth-newest leaves are present (Fig. 3). Xylem is located on the inside of the LVBs in unelongated nodes with large tubes, and the phloem is on the outside. Figure 3a is a vertical schematic of unelongated nodes, midveins, and LVBs of the first-through fourth-newest leaves (m1–m3, L4). We generated an upper horizontal section (b) and a lower section (f), as shown in Fig. 3a. Figure 3b is an image of section b, and Fig. 3c is a schematic of section b showing the LVBs, which are connected to the newest (red circles) and second-newest leaves (black circles). In the upper section b, expression was detected in the phloem of LVBs from the newest and second-newest leaves (m1 and m2 in Fig. 3b). Magnified views of m1 and m2 reveal expression mainly in the phloem of the newest leaf (Fig. 3e) and the second-newest leaf (Fig. 3d). In the lower section f, LVBs of the fourth-newest leaf formed extended LVBs (red circles in Fig. 3f) with strong GUS activity. Expression was detected mainly in the xylem of extended LVBs from the fourth-newest leaf (L4 in red in Fig. 3g). Supplementary Fig. 2 compares LVBs from the first- to the fifth-newest leaves in unelongated nodes. Expression was detected primarily in the phloem of new leaves (first and second; supplementary Fig. 2a, b) and around the xylem of old leaves (third to fifth; supplementary Fig. 2c–e).

### Gene expression and early growth of OsYSL16-knockdown plants in the germination stage

**Fig. 4:**
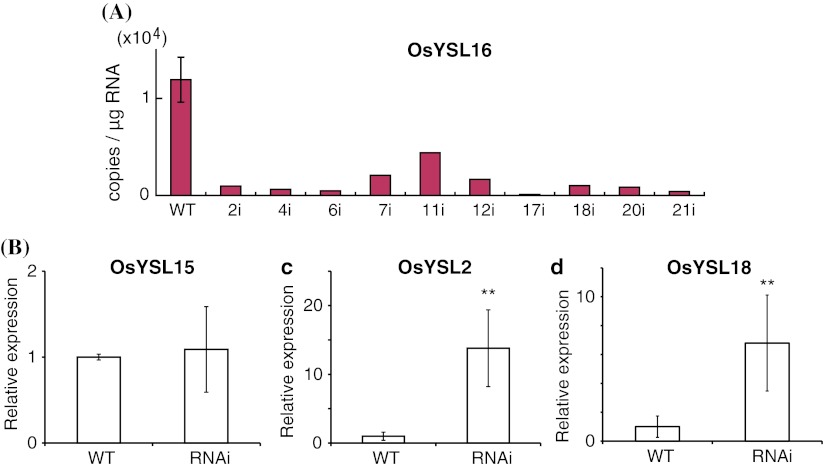
Gene expression in *OsYSL16*-knockdown plants. **a** Gene expression of *OsYSL16* in wild-type rice (WT) and *OsYSL16*-knockdown seedlings (2i, 4i, 6i, 7i, 11i, 12i, 17i, 18i, 20i, 21i). **b** Relative expression of *OsYSL15* in *OsYSL16*-knockdown seedlings (RNAi; average of expression in 17i, 20i and 21i) compared with WT. **c** Relative expression of *OsYSL2*. **d** Relative expression of *OsYSL18*. **Shows significant differences from WT (*p* < 0.01) by Student’s *t* test. Mean ± SD (*n* = 3)

**Fig. 5:**
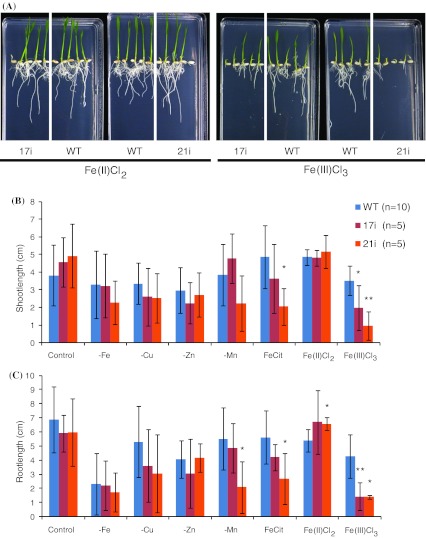
Early growth of *OsYSL16*-knockdown plants. **a** Picture of wild-type rice (WT) and *OsYSL16*-knockdown seedlings (17i, 21i) grown with different Fe sources. **b** Shoot length of wild-type plants (WT) and knockdown plants (17i and 21i) on MS medium with different metal nutrition conditions. **c** Root length. Fe(II)Cl_2_: 10 μM Fe(II)Cl_2_ in place of 100 μM Fe(III)-EDTA. Fe(III)Cl_3_: 10 μM Fe(III)Cl_3_ in place of 100 μM Fe(III)-EDTA. –Fe: MS medium without Fe. –Cu: MS medium without Cu. –Zn: MS medium without Zn. –Mn: MS medium without Mn. FeCit: 100 μM Fe(III)-citrate in place of 100 μM Fe(III)-EDTA. *Shows significant differences from WT (*p* < 0.05) based by Student’s *t* test (*n* of WT = 10, *n* of 17i and 21i = 5); mean ± SD, **Shows significant differences from WT (*p* < 0.01) by Student’s *t* test. The plants which did not germinate (when length of root or shoot was less than 1 mm) were not included in the calculation for statistics

We utilized RNA interference technique to knockdown *OsYSL16* expression in rice plants. The expression of *OsYSL16* in the transgenic plant seedlings was decreased by 70–99 % compared with expression in wild-type plants (WT; Fig. 4a). *OsYSL15* expression in knockdown plants was similar to that in wild-type plants (Fig. 4b). Expression levels of *OsYSL2* and *OsYSL18* in knockdown plants were 14 and six times the levels in wild-type plants on average, respectively (Fig. 4c, d). The early growth of *OsYSL16*-knockdown plants did not differ from that of wild-type plants on control MS medium and MS medium without Fe, Zn, Cu, or Mn (Fig. 5). When the Fe source was switched to 10 μM Fe(III)Cl_3_, however, the early growth of *OsYSL16*-knockdown plants was arrested (Fig. 5). This growth inhibition was not observed in medium containing 10 μM Fe(II)Cl_2_ (Fig. 5).

### Growth and Fe allocation of OsYSL16-knockdown plants in the vegetative stage

**Fig. 6:**
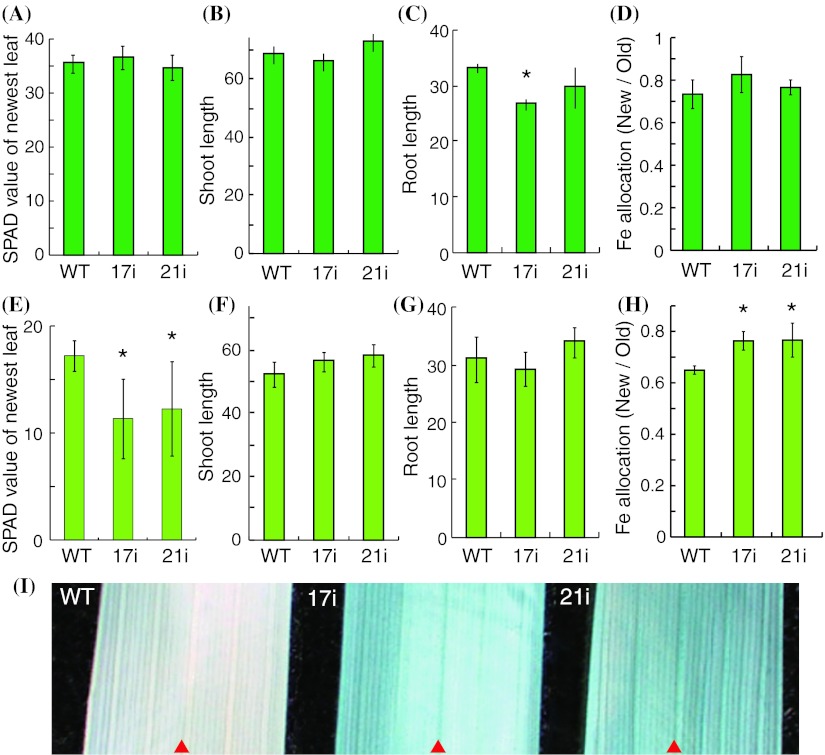
Growth of *OsYSL16*-knockdown plants in vegetative stage. Wild-type and knockdown (17i and 21i) plants after 4 (**a**–**c** and **e**–**g**) and 6 (**d**, **h**) weeks in a hydroponic solution containing 100 μM of Fe(III)-EDTA (**a**–**d**) and without Fe (**e**–**h**) for 1 week. **a** SPAD value under Fe-sufficient conditions. **b** Shoot length under Fe-sufficient conditions. **c** Root length under Fe-sufficient conditions. **d** Fe allocation into new (first- and second-newest) and old leaves (third-, fourth-, and fifth-newest) under Fe-sufficient conditions. **e** SPAD value under Fe-deficient conditions. **f** Shoot length under Fe-deficient conditions. **g** Root length under Fe-deficient conditions. **h** Fe allocation into new and old leaves under Fe-deficient conditions. *Shows significant differences from WT (*p* < 0.05) based by Student’s *t* test (*n* of 4-week-old WT = 10; *n* of 4-week-old 17i and 21i = 5; *n* of 6-week-old plants = 3); means ± SD

At 4 weeks of growth, the chlorophyll level (SPAD value) and shoot and root growth did not differ remarkably between *OsYSL16*-knockdown plants and wild-type plants in the presence of 100 μM Fe(III)-EDTA (Fig. 6a–c). No significant difference in shoot length was observed between knockdown and wild-type plants, and only one knockdown plant (17i) showed a shorter root length. Fe allocation into new (first- and second- newest) and old (third-, fourth- and fifth-newest) leaves was not different between wild-type and knockdown plants under Fe-sufficient conditions (Fig. 6d). When the plants were grown for 1 week without Fe, the SPAD value of the *OsYSL16*-knockdown plants was lower than that of the wild-type plants (Fig. 6e–g). The differences in SPAD value and size of plants between wild-type and knockdown lines at 6 weeks were similar to those at 4 weeks (data not shown). Under Fe-deficient conditions, Fe allocation into new leaves was significantly higher in knockdown plants than in wild-type plants (Fig. 6h). Fe allocation into roots, shoots, leaf blades, and leaf sheaths did not differ remarkably between knockdown and wild-type plants under both Fe-sufficient and Fe-deficient conditions (supplementary Fig. 3, 4). The Fe concentration in shoots was higher only in one knockdown plant (17i; supplementary Fig. 3b), and that in unelongated nodes was lower in knockdown plants than in wild-type plants under Fe-sufficient conditions (non-significantly in 17i and significantly in 21i; supplementary Fig. 3a). Fe distribution in leaves was analyzed by Prussian blue staining (Fig. 6i). In the most expanded leaves, only slight staining for Fe was observed in the veins of wild-type plants. In contrast, the *OsYSL16*-knockdown plants showed much enhanced accumulation of Fe in their veins (Fig. 6i).

**Fig. 7:**
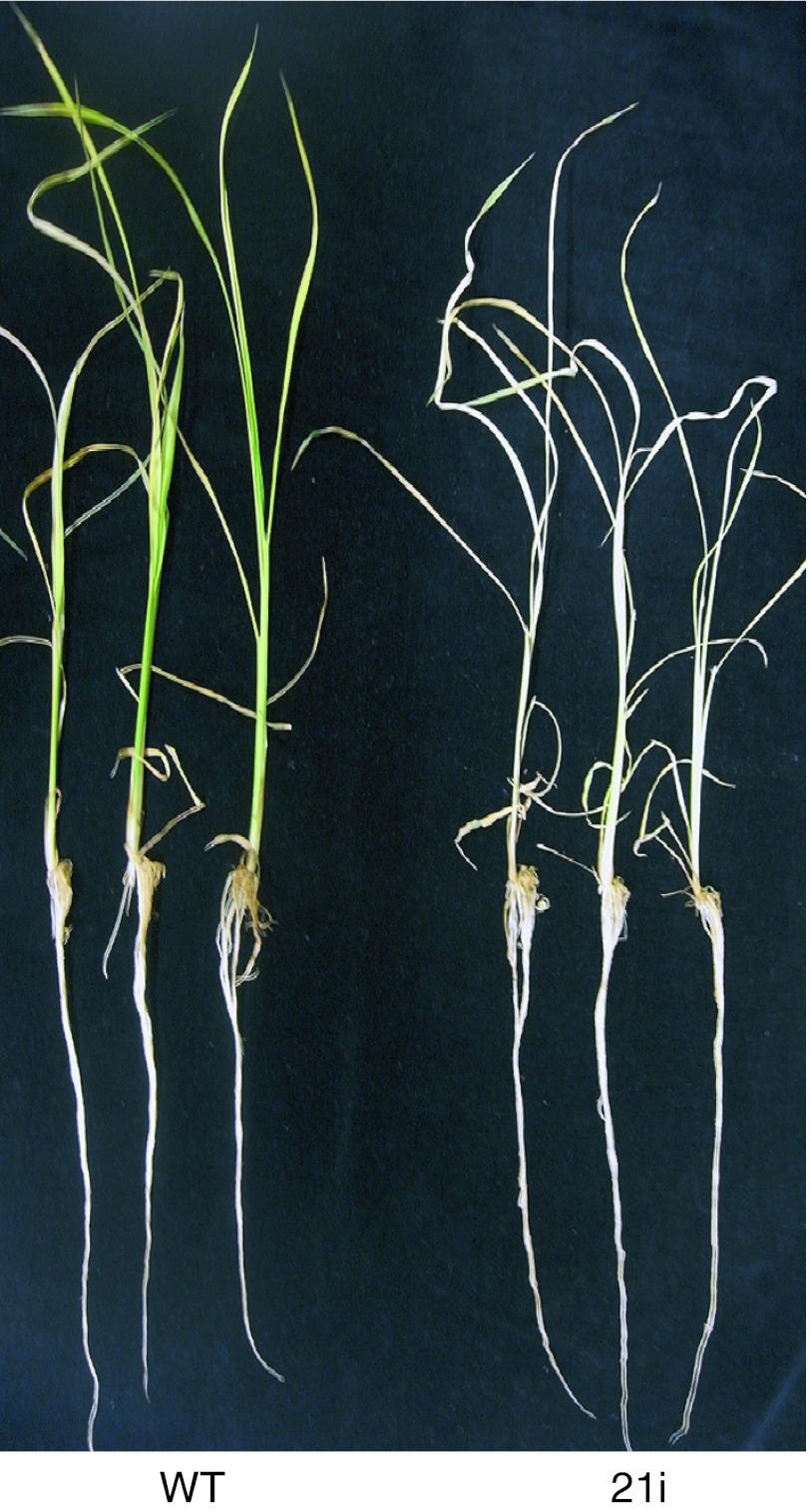
Picture of wild-type and *OsYSL16*-knockdown plants under prolonged Fe deficiency. Picture of 7-week-old wild-type and *OsYSL16*-knockdown (21i) plants grown in hydroponic culture without Fe for 3 weeks

When the knockdown plants were hydroponically grown for 3 weeks without Fe, the leaves became almost white, and the plants died; wild-type plant leaves remained yellow and alive (Fig. 7).

## Discussion

Rice OsYSL15, an Fe(III)-DMA transporter, takes up Fe from the rhizosphere (Inoue et al. 2009; Lee et al. 2009). OsYSL2, another YSL family transporter in rice (Koike et al. 2004), transports Fe(II)-NA and Mn(II)-NA, and is important for the translocation of Fe and Mn via the phloem (Ishimaru et al. 2010). The expression of these two YSL transporters is strongly induced under Fe-deficient conditions. Rice has two more genes, *OsYSL9* and *OsYSL16*, belonging to the same subgroup with *OsYSL15* and *OsYSL2* (Koike et al. 2004). OsYSL16 has 85 % homology to both OsYSL2 (75 % identity) and OsYSL15 (72 % identity). The OsYSL16-GFP fusion protein was localized to the cell plasma membrane (supplementary Fig. 1; Lee et al. 2012), as were OsYSL2 and OsYSL15 (Koike et al. 2004; Inoue et al. 2009; Lee et al. 2009). The yeast complementation assay demonstrated that OsYSL16 transports Fe(III)-DMA, but does not transport Fe(II)-NA (Fig. 1). This coincided with the transport activity of OsYSL15. Tissue localization of *OsYSL16* in roots and leaves (Fig. 2) was similar to the localization of the combination of *OsYSL2*, which is dominant in vascular bundles (Koike et al. 2004), and *OsYSL15*, which is dominant in the root epidermis (Inoue et al. 2009; Lee et al. 2009). *OsYSL16* is expressed in the root epidermis under both Fe-sufficient and Fe-deficient conditions (Fig. 2h, i), whereas *OsYSL15* expression is not detected under Fe-sufficient conditions (Inoue et al. 2009; Lee et al. 2009). This suggests that OsYSL16, like OsYSL15, takes up Fe(III)-DMA from the rhizosphere, especially when Fe is available in sufficient amounts. Lee et al. (2012) did not observe *OsYSL16* promoter-GUS expression in root epidermis, presumably because of difference in the position and growth stage of the observed samples. Supporting our results, RiceXPro public database for rice microarray analysis (http://ricexpro.dna.affrc.go.jp/index.html) also indicated that *OsYSL16* (Os04g0542800) was more strongly expressed in root epidermis/exodermis/sclerenchyma than in inner tissues.

*OsYSL16* was also expressed mainly around leaf xylem vessels (Fig. 2a, b), whereas *OsYSL2* was expressed mainly in the phloem (Koike et al. 2004). Similarly, *OsYSL16* was expressed around xylem vessels at the basal part of old leaves (supplementary Fig. 2e), whereas another Fe(III)-DMA transporter gene, *OsYSL18*, was expressed around the phloem (Aoyama et al. 2009). This difference suggests that the function of OsYSL16 in the leaf is mainly limited to translocation of Fe via the xylem, whereas OsYSL2 and OsYSL18 are specialized to translocate Fe via the phloem. In the xylem, Fe(III) is considered to be transported mainly with citrate (Yokosho et al. 2009). The DMA concentration in the xylem sap of rice increases under Fe-deficient conditions (Kakei et al. 2009). These findings suggest that DMA is secreted into the xylem, where it chelates some Fe(III) from Fe(III)-citrate complexes, and then OsYSL16 unloads Fe(III)-DMA from the xylem to allocate Fe to nearby cells.

The unelongated node is important for the translocation of minerals from roots to shoots. Tsukamoto et al. (2009) showed that Fe primarily accumulates at the basal part of stems with unelongated nodes and internodes, and then translocates to the leaves. The same author also reported that graminaceous plants translocate Fe from roots to old leaves mainly via the xylem, and to new leaves mainly via the phloem. Interestingly, inspection of *OsYSL16* promoter-GUS expression in unelongated nodes in the present study revealed strong expression of *OsYSL16* (Fig. 2c). Furthermore, when we observed LVBs connected to new leaves, *OsYSL16* promoter-GUS was expressed mainly in the phloem of unelongated nodes (Fig. 3, supplementary Fig. 2a, b). In contrast, in LVBs connected to old leaves, expression was observed mainly around xylem vessels (Fig. 3, supplementary Fig. 2c–e). Therefore, it is possible that OsYSL16 is responsible for the allocation of Fe to new and old leaves in unelongated nodes.

Lee et al. (2012) reported that activation of *OsYSL16* expression in rice results in enhanced Fe translocation from roots and germinating seeds to shoots. Generally, physiological function of a gene could be characterized more clearly by using gene knockout or knockdown, because gene enhancement can cause ectopic or secondary effect. Lee et al. (2012) also utilized knockout (T-DNA insertion) and knockdown (antisense) lines of *OsYSL16* and described that these plants showed no remarkable phenotypes. We applied another approach, RNA interference, to knockdown *OsYSL16* gene. Our *OsYSL16*-knockdown plants also showed very similar growth compared with wild-type in most of the growth conditions tested. Only when the Fe source in MS medium was replaced by Fe(III)Cl_3_ during early growth, however, *OsYSL16*–knockdown seedlings showed much impaired growth (Fig. 5). When the Fe source was replaced by Fe(II)Cl_2_, on the other hand, the growth of knockdown plants at the germination stage was similar to that of wild-type plants (Fig. 5). Clearly, *OsYSL16*–knockdown plants have an inferior ability to utilize Fe(III). Although Fe(II) can be taken up by OsIRT1 in rice roots, Fe(III) cannot be directly taken up unless chelated by MAs, because of low ferric-chelate reductase activity in rice roots (Ishimaru et al. 2006). Rice secretes DMA among MAs. Therefore, our results suggest that *OsYSL16*-knockdown plants did not efficiently utilize the Fe(III)-DMA complex. In the vegetative stage, the Fe concentrations in shoots and roots and plant growth did not differ markedly between knockdown plants and wild-type plants in hydroponic culture, both with and without Fe (Fig. 6, supplementary Fig. 3, 4). This indicates that the total amount of Fe taken up and translocated from roots to shoots was largely unaffected. Nevertheless, knockdown plants showed more severe chlorosis than wild-type plants when grown in hydroponic culture for 1 week without Fe (Fig. 6e), and died after 3 week-culture without Fe (Fig. 7). Notably, Fe accumulated in the veins of *OsYSL16*-knockdown plants (Fig. 6i). These results suggest that OsYSL16 allocates Fe(III)-DMA from the xylem to nearby cells, facilitating proper Fe distribution and homeostasis.

*OsYSL16*-knockdown plants unexpectedly allocated more Fe into new leaves under Fe-deficient conditions (Fig. 6h). This might be because of increased demand of Fe translocation into young leaves which was caused by disrupted Fe distribution and severer Fe deficiency, which might have stimulated the expression of other Fe transporters. Indeed, *OsYSL2* and *OsYSL18* were more highly expressed in knockdown plants (Fig. 4c, d). This result also raises the possibility that the allocation of Fe into new leaves via phloem vessels was complemented partially by highly expressed OsYSL2, and similarly that the allocation into old leaves was complemented partially by OsYSL18. On the other hand, enhanced expression of *OsYSL2* and *OsYSL18* is less likely to be attributed to Fe-inefficient phenotype of the *OsYSL16*-knockdown plants. In particular, expression of *OsYSL2* is induced under Fe deficiency (Koike et al. 2004). Therefore, enhanced expression of *OsYSL2* would result in enhanced Fe efficiency rather than Fe inefficiency, unless its localization is much disrupted (Ishimaru et al. 2010). Total Fe content, as well as *OsYSL15* expression, was not decreased in *OsYSL16*-knockdown plants (Fig. 4b; supplementary Fig. 3, 4), suggesting that the amount of Fe to be taken up by OsYSL16 is not a dominant proportion of the total amount of Fe, including Fe(II), taken up by many Fe-transporters. Many plasma membrane-localized Fe transporters have been identified in rice; these include OsYSL2, 15, 16, and 18; OsIRT1; and OsNRAMP1 (Aoyama et al. 2009; Curie et al. 2000; Inoue et al. 2009; Ishimaru et al. 2006, 2010; Lee et al. 2009; Koike et al. 2004; Takahashi et al. 2011). These transporters function cooperatively and can functionally complement each other when one is impaired.

## Electronic supplementary material

Below is the link to the electronic supplementary material.

Supplementary material 1 (PDF 353 kb)
