# Supplementary material for: OsYSL16 plays a role in the allocation of iron
Source: Plant Mol Biol. 2012 May 29;79(6):583–94. doi: 10.1007/s11103-012-9930-1 (PMC3402674; doi:10.1007/s11103-012-9930-1)
Supplement: Supplementary file 1 — Supplementary material 1 (PDF 353 kb) [file 11103_2012_9930_MOESM1_ESM.pdf]

a b c d

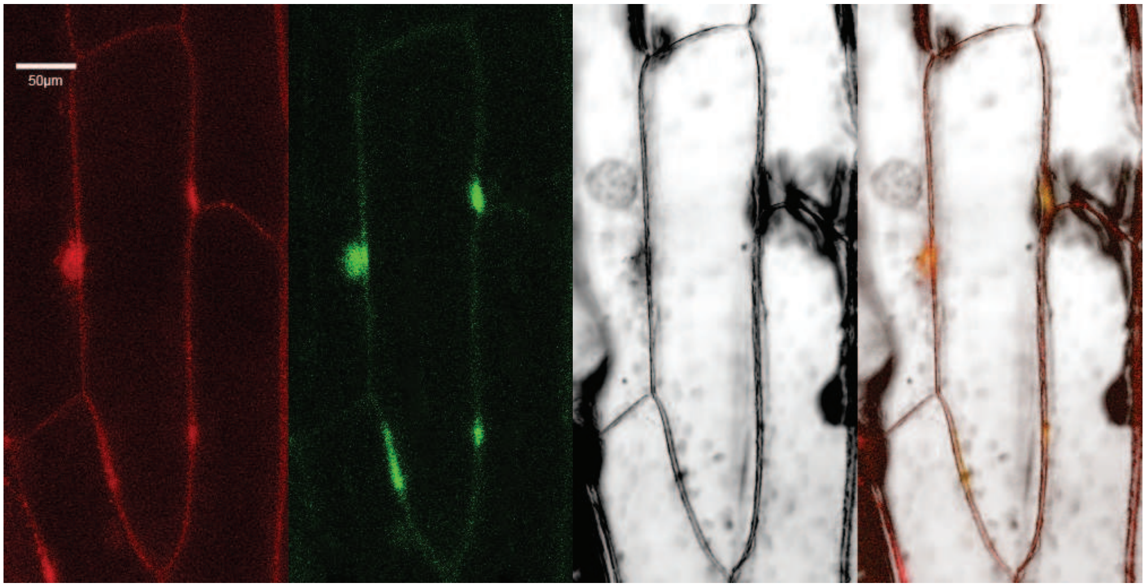

Supplementary Fig. 1 Subcellular localization of OsYSL16

OsYSL16 fused with GFP was expressed in onion epidermal cells. FM4-64 (2 μM) was used as a plasma membrane marker. a. FM4-64. b. Fluorescence image. c. Bright field image. d. Merged image. Scale bar is 50 μm long.

Article title:OsYSL16 plays a role in the allocation of iron, Journal name:Plant Mol Biol, Author names:Yusuke Kakei, Yasuhiro Ishimaru, Takanori Kobayashi, Takashi Yamakawa, Hiromi Nakanishi, Naoko K Nishizawa, Affiliation of corresponding author:1.Graduate School of Agricultural and Life Sciences, The University of Tokyo, 1-1-1 Yayoi, Bunkyo-ku, Tokyo 113-8657, Japan, 2.Research Institute for Bioresources and Biotechnology, Ishikawa Prefectural University, 1-308 Suematsu, Nonoichi-machi, Ishikawa 921-8836, Japan, E-mail: annaoko@mail.ecc.u-tokyo.ac.jp

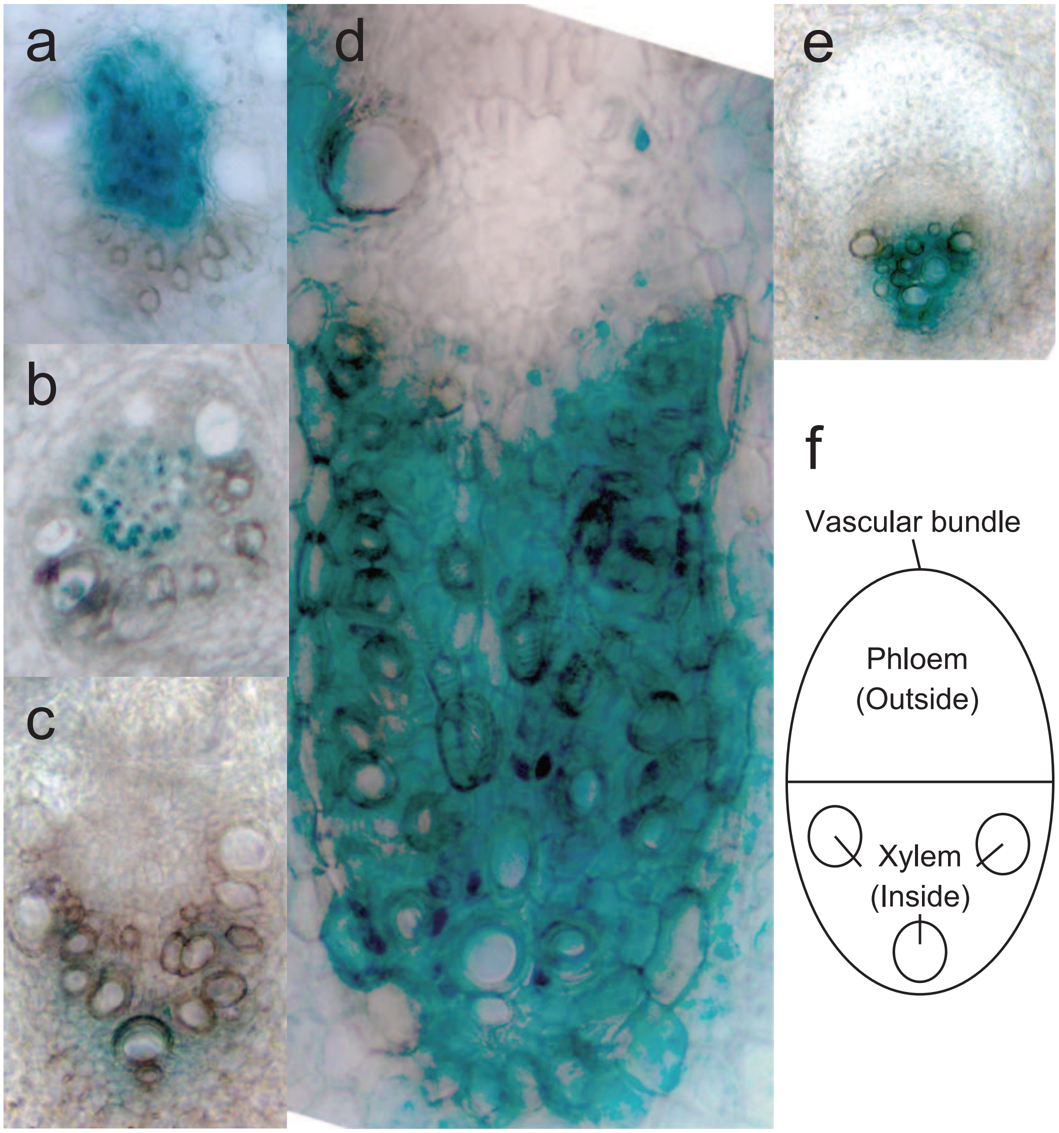

Supplementary Fig. 2 Magnified images of large vascular bundles in unelongated nodes

a.Large vascular bundle (LVB) of the newest leaf in unelongated node. b. LVB of second-newest leaf. c. LVB of third-newest leaf. d. LVB of fourth-newest leaf. e. LVB of fifth newest leaf at the basal part of leaf sheath. f. A schematic of phloem and xylem in vascular bundle of unelongated nodes.

Article title:OsYSL16 plays a role in the allocation of iron, Journal name:Plant Mol Biol, Author names:Yusuke Kakei, Yasuhiro Ishimaru, Takanori Kobayashi, Takashi Yamakawa, Hiromi Nakanishi, Naoko K Nishizawa, Affiliation of corresponding author:1.Graduate School of Agricultural and Life Sciences, The University of Tokyo, 1-1-1 Yayoi, Bunkyo-ku, Tokyo 113-8657, Japan, 2.Research Institute for Bioresources and Biotechnology, Ishikawa Prefectural University, 1-308 Suematsu, Nonoichi-machi, Ishikawa 921-8836, Japan, E-mail: annaoko@mail.ecc.u-tokyo.ac.jp

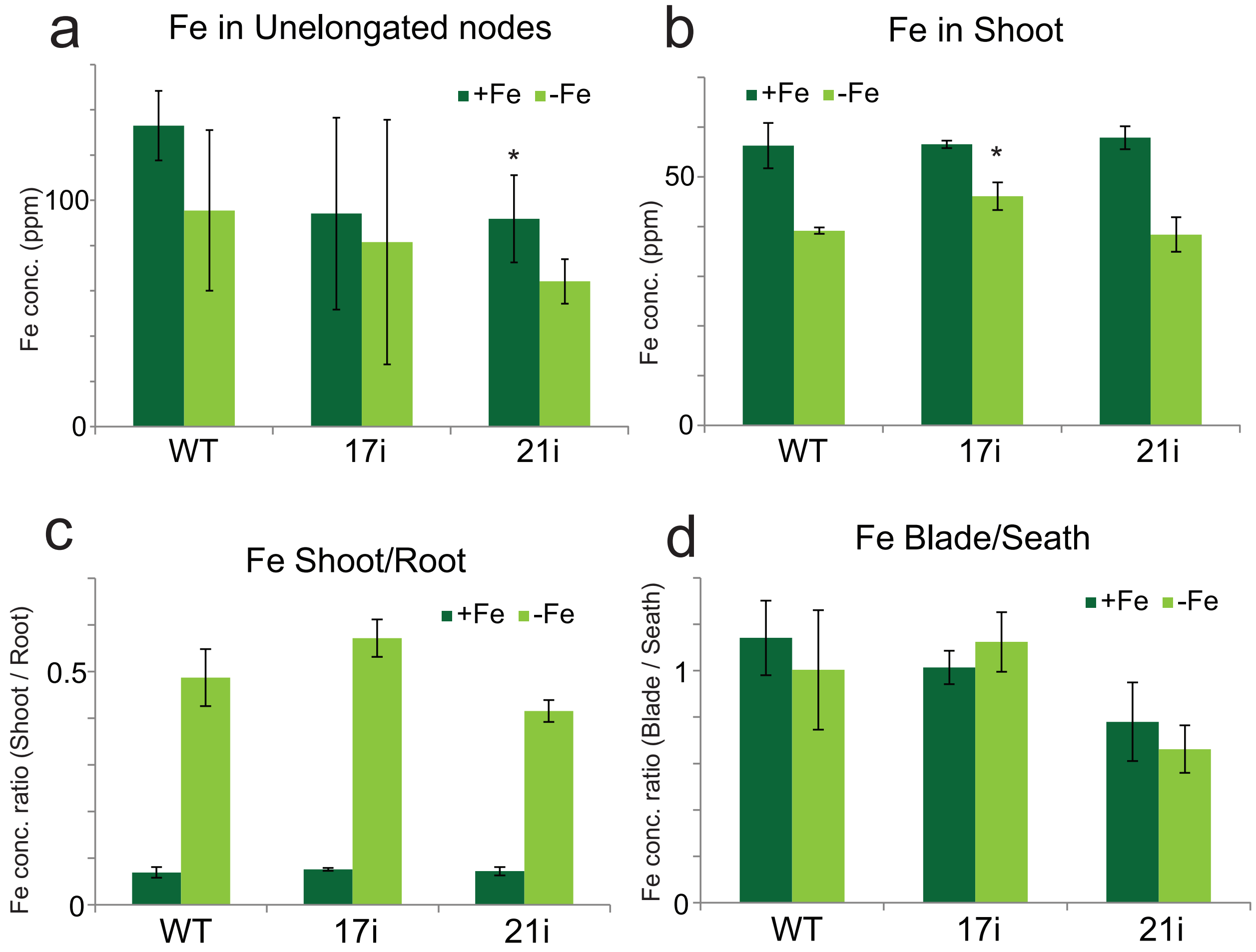

Supplementary Fig 3 Iron concentration and allocation in *OsYSL16*-knockdown plants

Wild-type plants (WT) and knockdown plants (17i and 21i) after 6 weeks in hydroponic solution containing 100 μM of Fe(III)-EDTA (+Fe) and without Fe (-Fe) for 1 week were harvested, dissected into tissues, and subjected to Fe concentration measurement. a. Iron concentration in unelongated nodes. b. Iron concentration in shoot. c. Iron allocation into shoot and root. d. Iron allocation into leaf blades and sheaths. * shows significant differences from WT (p < 0.05) based by Student' s *t*-test (n = 3); means ± SD.

Article title:OsYSL16 plays a role in the allocation of iron, Journal name:Plant Mol Biol, Author names:Yusuke Kakei, Yasuhiro Ishimaru, Takanori Kobayashi, Takashi Yamakawa, Hiromi Nakanishi, Naoko K Nishizawa, Affiliation of corresponding author:1.Graduate School of Agricultural and Life Sciences, The University of Tokyo, 1-1-1 Yayoi, Bunkyo-ku, Tokyo 113-8657, Japan, 2.Research Institute for Bioresources and Biotechnology, Ishikawa Prefectural University, 1-308 Suematsu, Nonoichi-machi, Ishikawa 921-8836, Japan, E-mail: annaoko@mail.ecc.u-tokyo.ac.jp

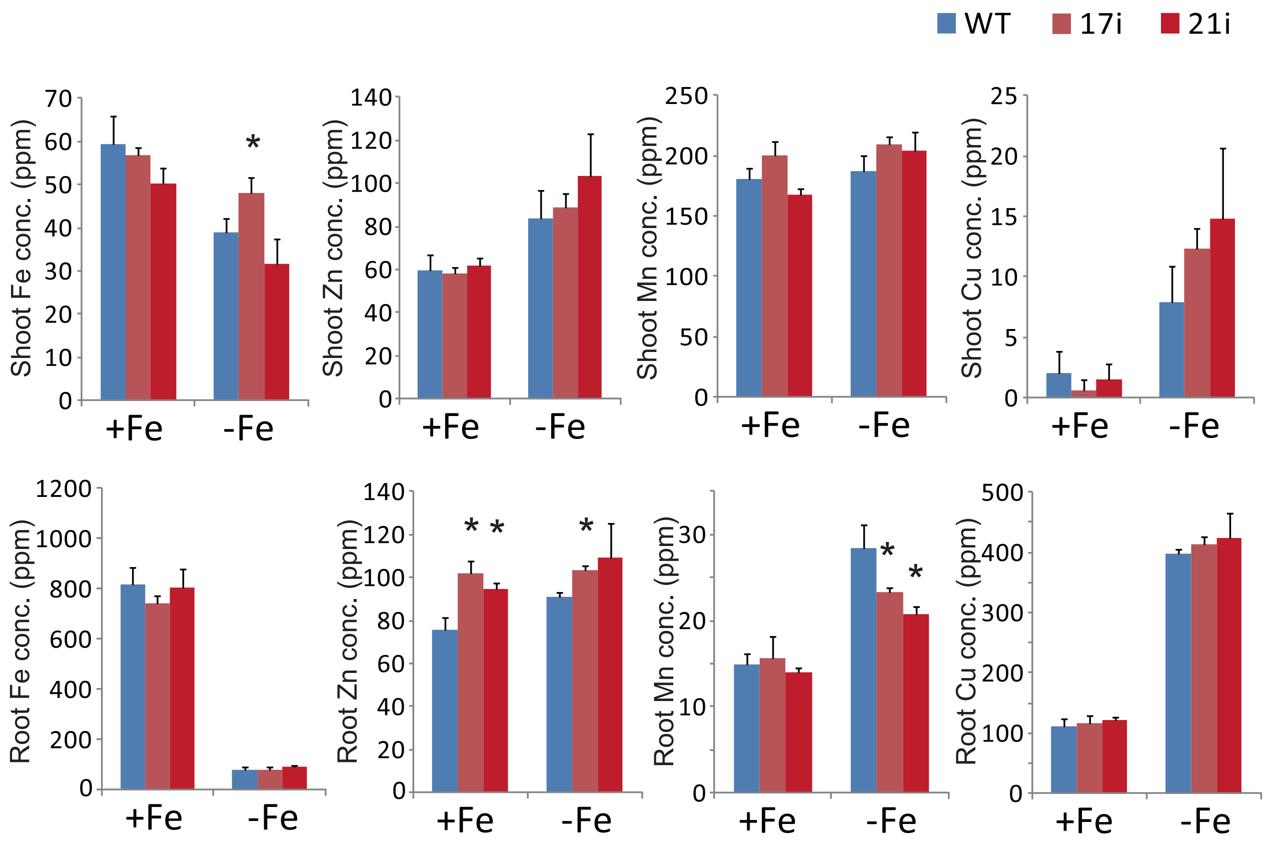

Supplementary Fig 4 Metal concentrations in *OsYSL16*-knockdown plants

Metal concentrations of wild-type (WT) and *OsYSL16*-knockdown plants (17i, 21i) were analyzed using 6 week-old plants grown in Fe sufficient (+Fe) and deficient (-Fe) hydroponic cultures for 1 week. * shows significant differences from WT (p < 0.05) based by Student' s *t*-test (n = 3); means ± SD (n = 3).

Article title:OsYSL16 plays a role in the allocation of iron, Journal name:Plant Mol Biol, Author names:Yusuke Kakei, Yasuhiro Ishimaru, Takanori Kobayashi, Takashi Yamakawa, Hiromi Nakanishi, Naoko K Nishizawa, Affiliation of corresponding author:1.Graduate School of Agricultural and Life Sciences, The University of Tokyo, 1-1-1 Yayoi, Bunkyo-ku, Tokyo 113-8657, Japan, 2.Research Institute for Bioresources and Biotechnology, Ishikawa Prefectural University, 1-308 Suematsu, Nonoichi-machi, Ishikawa 921-8836, Japan, E-mail: annaoko@mail.ecc.u-tokyo.ac.jp
